# Robust Intrapulmonary CD8 T Cell Responses and Protection with an Attenuated N1L Deleted Vaccinia Virus

**DOI:** 10.1371/journal.pone.0003323

**Published:** 2008-10-02

**Authors:** Anuja Mathew, Joel O'Bryan, William Marshall, Girish J. Kotwal, Masanori Terajima, Sharone Green, Alan L. Rothman, Francis A. Ennis

**Affiliations:** 1 Center for Infectious Disease and Vaccine Research, University of Massachusetts Medical School, Worcester, Massachusetts, United States of America; 2 Department of Medicine, University of Massachusetts Medical School, Worcester, Massachusetts, United States of America; AIDS Research Center, Chinese Academy of Medical Sciences and Peking Union Medical College, China

## Abstract

**Background:**

Vaccinia viruses have been used as a model for viral disease and as a protective live vaccine.

**Methodology and Principal Findings:**

We investigated the immunogenicity of an attenuated strain of vaccinia virus engineered to inactivate the N1L gene (vGK5). Using the intranasal route, this recombinant virus was 2 logs less virulent compared to the wildtype VACV-WR. Infection by the intranasal, intraperitoneal, and tail scarification routes resulted in the robust induction of cytolytic virus-specific CD8 T cells in the spleens and the lungs. VACV-specific antibodies were also detected in the sera of mice infected 3–5 months prior with the attenuated vGK5 virus. Finally, mice immunized with vGK5 were significantly protected when challenged with a lethal dose of VACV-WR.

**Conclusions:**

These results indicate that the attenuated vGK5 virus protects against subsequent infection and suggest that the N1L protein limits the strength of the early antiviral CD8 T cell response following respiratory infection.

## Introduction

Vaccinia virus (VACV) is a member of the Poxviridae family and is well established as a model for the study of poxvirus biology and disease, with the route of administration playing a major role in outcome. VACV has also been studied as recombinant vector to express foreign genes for human immunotherapy and as a vaccine vector against a variety of diseases. Strains of vaccinia virus which are in the licensed smallpox vaccines have played a central role in the eradication of smallpox. Smallpox was eradicated in 1977, but after September 11, 2001 there has been renewed interest in variola virus (the etiologic agent for smallpox) and its potential use as a bioweapon. The relatively high incidence of adverse events in immunocompetent individuals following immunization with the currently licensed vaccine (NYCBH virus/DryVax) [1], such as myocarditis and eczema vaccinatum, has been a recent deterrent to immunizing the general population [2].

Although mice are not a natural host for VACV, murine models of VACV have been used to assess the efficacy of candidate vaccines and define strategies for immune protection. The Western Reserve strain of VACV (VACV-WR) is relatively more pathogenic for mice than other strains of VACV, due to its previous adaptation to grow in mouse brain [3]. I.p. immunization of mice with VACV-WR induces robust T cell responses [4]. Intranasal (i.n.) infection with VACV-WR simulates the spread of smallpox virus throughout the respiratory tract with subsequent spread to other visceral organs such as the ovaries, spleen, lungs, and liver [5]–[7], but mice develop a low primary immune response. Approaches that promote effective pulmonary T cell mediated immunity are therefore important to evaluate T cell response in mucosal sites where they serve as an important line of defense following respiratory challenge with smallpox virus.

VACV encode for many proteins that modulate aspects of host immunity [8]–[12]. For example the A44L protein of VACV is immunosuppressive and affects local and systemic steroid levels during VACV infection [11]. The N1L protein is a known virulence determinant of VACV [13], [14] that functions by targeting components of the IKK complex to inhibit NF-kB and IRF3 activation [15]. The identification of N1L as an inhibitor of both innate immune signaling and cytokine secretion [15], [16], implied that viruses deficient in the N1L gene would enhance vaccinia virus-specific adaptive T cell responses. Recently a novel function for the N1L protein as an antiapoptotic molecule has also been suggested by two groups [17], [18]. Preliminary studies of the adaptive immune response to the N1L-deficient virus were performed in balb/c mice immunized by tail scarification and by footpad inoculation [14] and prior to the identification of VACV-specific T cell epitopes. Intracranial injection of the N1L deficient virus, vGK5 provided protection against a subsequent challenge with VACV-WR [19]. While previous studies defined the attenuated VACV as significantly less pathogenic than the mouse adapted neurovirulent VACV-WR, the accompanying immune responses were not fully assessed [13]. Therefore it was not possible to determine the relationship of pathogenicity to immunogenicity and viral replication.

We hypothesized that the attenuated N1L-deficient VACV (vGK5) would be capable of initiating a robust T cell immune response following intranasal infection. To understand the dynamics of virus-specific CTL in detail, we used a tetramer against the immunodominant B8R_20–27_ epitope described in C57/BL6 mice to both track and phenotype antigen-specific T cells in the lungs and spleens of mice after infection [20]. Our results indicate that the recombinant attenuated vGK5 induced robust CD8+ T cell responses when administered by the intranasal, tail scarification, and systemic routes. Mice that were immunized with the attenuated virus by the respiratory route were also significantly protected from a lethal challenge with wildtype virus. Respiratory infection with vGK5 induced a better balance between immunogenicity and virulence than respiratory infection with the parent virus VACV-WR.

## Results

### Recombinant VACV engineered to lack the N1L gene are attenuated in C57/BL6 mice following intranasal infection

**Figure 1:**
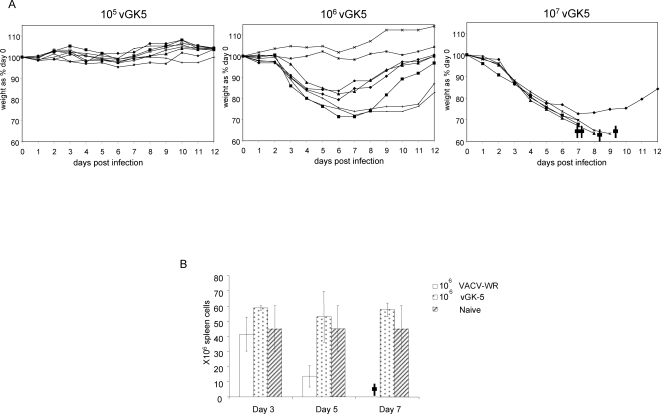
Weight loss curves of individual mice and splenocyte counts following i.n. VACV infection of C57/BL6 mice. (A) Groups of female C57/BL6 mice (n = 5–8) were infected with 10^5^, 10^6^ and 10^7^ PFU of vGK5 by the i.n. route. The percentage of weight relative to the initial body weight (100%) was plotted and the data are presented as percent change in body weight following infection. † depicts days that individual mice were last alive. (B) Average spleen counts±standard deviation of mice were assessed by trypan blue exclusion at days 3, 5 and 7 post infection with 10^6^ VACV-WR and vGK5 by the i.n. route. Data shown are representative of 2 experiments performed and demonstrate that high dose VACV-WR i.n. infections result in significantly (p<0.05) lower lymphocytes in the spleens during acute infection.

We first assessed the pathogenicity of the attenuated N1L deficient vGK5 virus and the wildtype VACV-WR after intranasal infection as this route of infection with VACV simulates smallpox infection in humans. In agreement with published data [6], [21]–[23], the LD50 for VACV-WR in C57/BL6 mice was 4.2×10^4^ PFU. Mice were next monitored for weight loss and survival after infection with varying doses of the recombinant attenuated virus, vGK5 virus (Fig. 1A). Lines represent weight curves of individual mice. The LD50 in age matched C57/BL6 mice was 4.2×10^6^ PFU based on the Reed and Muench method [24]. We determined the absolute numbers of lymphocytes recruited into the spleens of mice infected with 10^6^ PFU VACV-WR and vGK5 by the intranasal route. We detected a significant decrease in the total number of splenocytes in mice that were administered VACV-WR. By day 7, these mice were moribund (Fig. 1B). In contrast, when mice were administered 10^6^ PFU of the attenuated vGK5 virus by the i.n. route, there was an increase in the total number of lymphocytes in the spleens of infected mice with greater than 50% of the CD8 T cells expressing high levels of the activation marker CD11a (data not shown).

### Increased expansion and activation of B8R_20–27_ specific CD8 T cells following respiratory infection with the attenuated vGK virus

**Figure 2:**
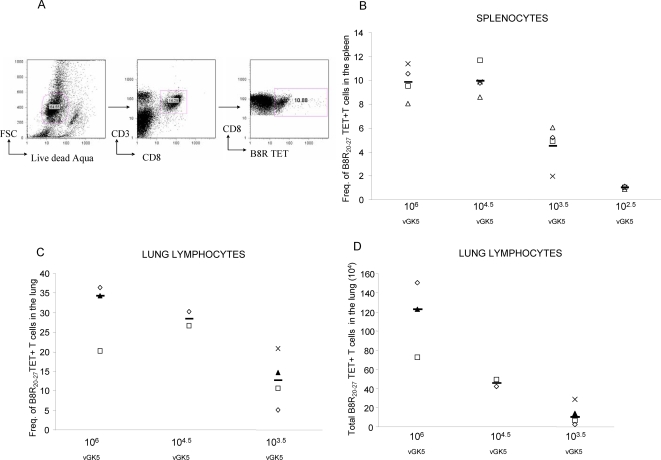
Tetramer frequencies following i.n. infection with vGK5. Mice were infected with varying doses of vGK5 by the i.n. route. (A) We used a gating strategy to identify live CD3^+^CD8^+^ T lymphocytes (live dead aqua negative and forward scatter positive; CD3+CD8+). Frequencies of B8R_20–27_ tetramer+ T cells were assessed in the (B) splenocytes and (C) lung lymphocytes 7 days after i.n. infection. (D) The absolute numbers of B8R_20–27_ tetramer+ T cells in the lung following i.n. infection. Each symbol represents the frequency of tetramer+ T cells obtained in target organs of individual mice; median values are denoted by horizontal lines.

In order to more carefully dissect out the immunogenicity of the attenuated N1L deleted VACV, we infected mice with varying sublethal doses of vGK5 (10^6^, 10^4.5^, 10^3.5^ and 10^2.5^) by the i.n. route. To measure frequencies of antigen-specific T cells in target organs, we obtained a tetramer directed against the immunodominant epitope B8R_20–27_ described in H-2b mice [20]. Using the gating strategy shown in Fig. 2A, the highest frequencies of tetramer+ T cells were detected in spleens of mice that were infected with 10^6^ and 10^4.5^ PFU of vGK5 (Fig. 2B). Frequencies of B8R_20–27_ -specific tetramer+T cells were lower in mice infected with 10^3.5^ PFU of vGK5 and near background levels with 10^2.5^ of vGK5. Lungs of mice infected with 10^6^ PFU of vGK5 also had the highest frequencies (Fig. 2C) as well as total number of tetramer+ T cells (Fig. 2D) compared to lungs of mice infected with lower doses of virus. All of the tetramer positive cells expressed high levels of the activation marker CD44 (data not shown). The data suggest that administration of the attenuated vGK5 by the i.n. route induced robust activation of CD8 T cells in mucosal and systemic sites.

### Robust VACV-specific T and B cell responses to intranasal vGK5 infection

**Figure 3:**
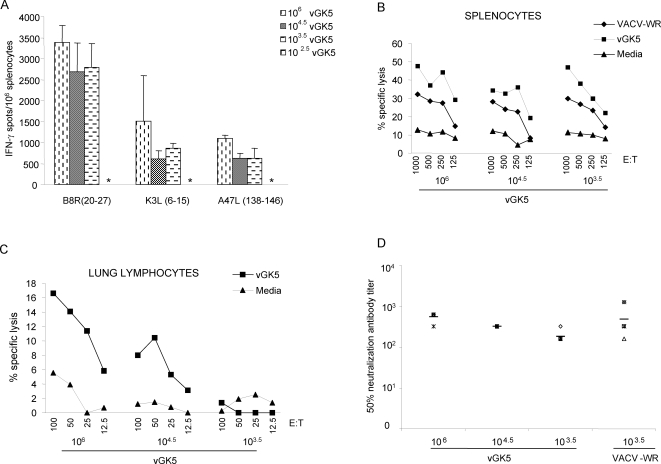
Cytokine responses and cytolytic activity in target organs. (A) IFN-γ responses of splenocytes from intranasally infected mice were measured in response to 3 VACV-specific CD8 T cell peptides (1 µg/ml) in an Elispot assay. Assays were performed using triplicate wells for each condition and individual mice/group. * = no responses detected. Seven days post infection, (B) splenocytes and (C) lung lymphocytes were isolated and CTL assays were carried out using RMA cells infected with VACV-WR (moi = 5), vGK5 (moi = 5) at different effector to target (E/T) ratios. Data shown are 1of 2 experiments performed. (D) PRNT50 antibody titers were measured in the sera of mice immunized 5 months prior with varying doses of vGK5 or 10^3.5^ PFU of VACV-WR (n = 5/group).

We assessed the ability of splenocytes from mice infected intranasally with different doses of vGK5 to secrete IFN-γ in response to the B8R_20–27_ peptide as well as two additional VACV-specific peptides K3L_6–15_ and A47L_138–146_ [20]. Frequencies of IFN-γ secreting cells to all 3 peptides were significantly higher in mice infected with 10^6^ PFU than lower doses of vGK5 (Fig. 3A). Similar responses were detected in mice infected with 10^3.5^ or 10^4.5^ PFU of vGK5 to all peptides. A cardinal property of activated CD8 T cells during acute viral infections is their ability to lyse and eliminate virus infected cells. Splenocytes and lung lymphocytes from mice infected by the i.n. route were therefore tested for their ability to lyse virus infected target cells in ex vivo CTL assays. Splenocytes from mice infected with 10^3.5^, 10^4.5^ and 10^6^ PFU of vGK5 all effectively lysed virus infected target cells (Fig. 3B). No CTL activity was detected in the spleens of mice infected with 10^2.5^ PFU of vGK5 (data not shown). CTL activity was only detected in lung lymphocytes of mice infected with 10^6^ and 10^4.5^ PFU of vGK5 by the i.n. route (Fig. 3C). Our data thus far indicated that T lymphocytes in the spleens and lungs of mice immunized with vGK5 were activated, elicited cytokine responses to VACV-specific peptides and had lytic activity against virus infected cells.

We next assessed VACV-specific antibody responses in mice immunized with varying doses of vGK5 by the i.n. route. Sera from mice immunized five months prior with 10^6^, 10^4.5^ and 10^3.5^ vGK5 had 50% PRNT neutralization titers which ranged from 160–640 (Fig. 3D) which were not significantly different from PRNT_50_ titers in the sera of mice immunized with 10^3.5^ VACV-WR. Our data indicate that the attenuated vGK5 elicited robust T cell as well as antibody responses in mice immunized by the i.n. route.

### Systemic infection and dermal scarification with vGK5 also results in robust adaptive immunity

**Figure 4:**
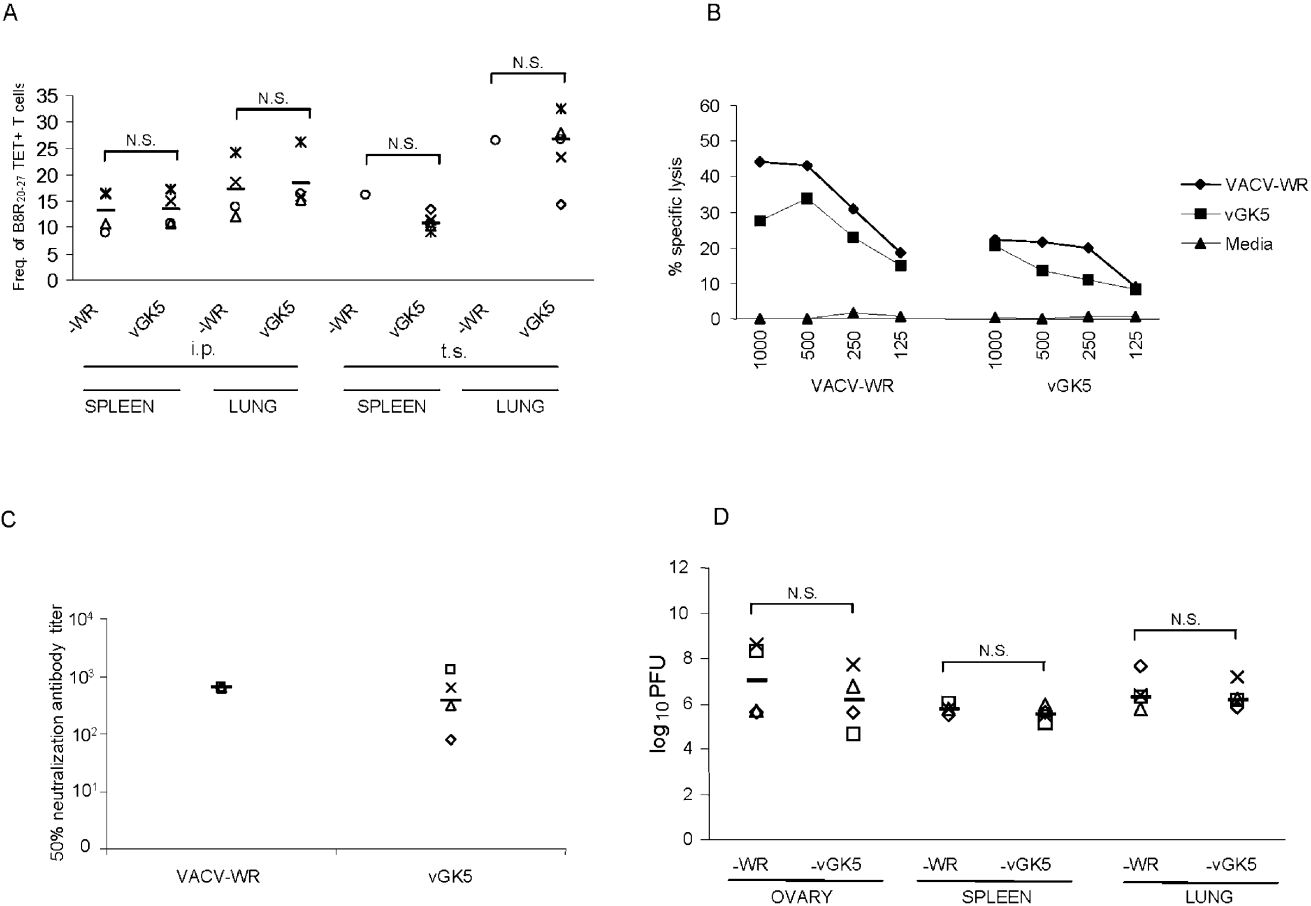
Immune responses following infection by the tail scarification and i.p. routes. Mice were infected with 1×10^6^ PFU VACV-WR or vGK5 by the i.p. and t.s. routes. (A) Lung lymphocytes and splenocytes obtained from mice (n = 4 mice/group except for infection with VACV-WR by the t.s. route where splenocytes and lung lymphocytes from 2 mice were pooled together) infected 7 days prior were stained with B8R_20–27_ tetramer. The data shown represent frequencies of cells that were tetramer positive within the CD3+CD8+ gate. Each symbol represents the frequency of tetramer+ T cells obtained in target organs of individual mice; median values are denoted by horizontal lines. (B) Seven days post infection, splenocytes were isolated and CTL assays were carried out using RMA cells infected with VACV-WR (moi = 5), vGK5 (moi = 5) at different (E/T) ratios. Data shown are representative of 2–3 experiments performed for each condition for the i.p. route. (C) PRNT50 antibody titers were measured in sera of mice immunized 3 months prior with 10^6^ PFU of VACV-WR (n = 3) or vGK5 (n = 4). (D) VACV titers were determined in organs 5 days post infection by the i.p. route and expressed as log_10_ PFU per gram of lung and spleen tissue and PFU/ovary. – represents median values of titers in respective organs. N.S. = Not significant. P values were determined by Student's *t* test.

Overall our studies thus far showed that mice could tolerate high doses of vGK5 by the i.n. route and these doses elicited robust CD8 T cell responses in the lungs and spleens of acutely infected mice. To determine whether immune responses to the attenuated vGK5 were comparable to wildtype VACV-WR, we administered equivalent doses of both viruses by the i.p. and tail scarification routes (10^6^ PFU) and lower doses by the i.n. route (10^3.5^ PFU) since mice were unable to tolerate 10^4^ or greater doses of wildtype VACV-WR intranasally. Mice that were administered VACV-WR or vGK5 by the i.p. or tail scarification routes did not lose any weight and remained healthy. Seven days post infection, 10% of the CD8+ T cells in the spleens and 13–16% of CD8+ T cells in the lungs of mice infected systemically with VACV-WR or vGK5 were tetramer positive with similar frequencies of B8R_20–27_ TET+ T cells detected in mice infected by the tail scarification route (Fig. 4A). Splenocytes from mice infected with vGK5 systemically efficiently lysed VACV-infected target cells although VACV-WR elicited slightly higher responses at all E/T ratios tested (Fig. 4B). To compare antibody titers in mice immunized with wildtype or the attenuated vGK5, we collected sera from mice immunized 3 months prior with 10^6^ PFU VACV-WR or vGK5 by the i.p. route. Sera from mice immunized with vGK5 had vaccinia-specific antibody titers ranging from 80–1280 (Geometric Mean Titer = 380) while sera from mice immunized with VACV-WR had PRNT_50_ titers of 640 (Fig. 4C). There were no statistical differences between the two groups.

At day 7 post infection, 5.4 (±1.7)% of CD8 T cells in the spleens of mice infected with 10^3.5^ VACV-WR i.n. were B8R_20–27_ specific which was similar to the frequencies in mice given 10^3.5^ vGK5 by the i.n. route (Fig. 2B). Frequencies of lung lymphocytes and elispot responses were also similar in mice administered 10^3.5^ PFU of both viruses (data not shown). Our data indicate that the attenuated vGK5 virus is immunogenic and elicits robust immune responses that are comparable to the wildtype VACV-WR when administered by multiple routes.

### Distribution and titers of VACV in target organs after intranasal and systemic infection

**Figure 5:**
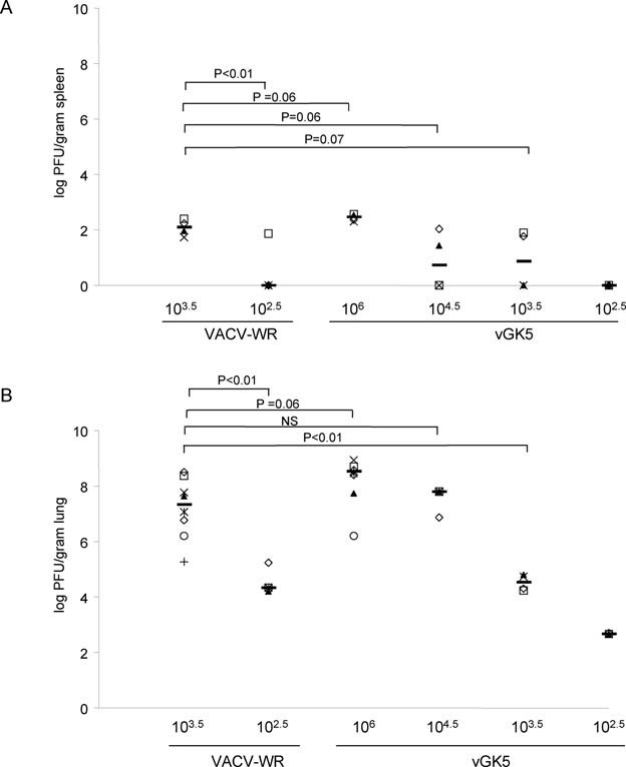
Viral titers following i.n. infections. (A) Spleens and (B) lungs (n = 4–8 per group) were harvested on day 7 from mice infected with VACV-WR and vGK5 by the i.n. route. VACV titers were determined and expressed as log_10_ PFU per gram of lung and spleen tissue. – represents median values of titers in respective organs. N.S. = not significant. Each symbol represents the titer obtained in target organs of individual mice; median values are denoted by horizontal lines. P values were determined by Student's *t* test.

Since the timing of antigen exposure has been shown to influence the magnitude and quality of the CD8+ T cell response, we examined viral replication in multiple organs including the spleens, lungs and ovaries of mice infected by the i.n. and i.p. route. 7 days post infection, viral titers were low but detectible in the spleens of mice infected with 10^6^, 10^4.5^ and 10^3.5^ vGK5 by the i.n. route (Fig. 5A) and titers were several logs higher in the lungs of these mice at the same time point (Fig. 5B). Viral titers were 3–4 logs higher in the lungs of mice administered 10^3.5^ VACV-WR compared to lungs of mice administered 10^3.5^ vGK5 (Fig. 5B). Equivalent viral loads were achieved in the lungs of mice infected with 10^4.5^ PFU of vGK5 and 10^3.5^ VACV-WR 7 days post infection. On day 5, mice that were infected via the i.p. route with both viruses had similar titers of virus in the ovaries, lungs and spleens (Fig. 4D). By day 7, virus was cleared from both the lungs and spleens of mice infected by the i.p. route while titers in the ovaries were detectible but at similar levels (data not shown).

These data indicate that the attenuated vGK5 virus replicates to high levels in target organs. The route of inoculation influences the level of replication between the attenuated vGK5 virus and wildtype VACV-WR when similar doses are administered.

### Mice immunized with vGK5 are protected from a lethal respiratory challenge with VACV-WR

**Figure 6:**
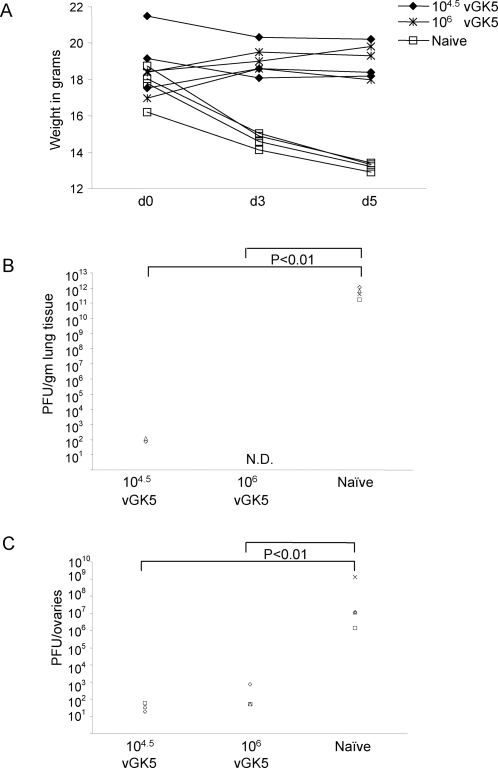
Mice immunized with vGK5 are protected from a lethal challenge with VACV-WR. Mice were infected with 10^4.5^, 10^6^ PFU of vGK5 (n = 3/group) by the intranasal route. 1 month later immunized mice and age matched naïve controls were challenged with a lethal dose of VACV-WR (10^6^ PFU) by the i.n. route. (A) Weights of mice were monitored over 5 days. Viral titers were measured in the (B) lungs and (C) ovaries and expressed as viral titers/gm lung tissue or ovaries. Each symbol represents the titer obtained in target organs of individual mice.

Finally, to determine whether vGK5 infected mice were protected from a lethal challenge with the neurovirulent VACV-WR, mice that were immunized with 10^4.5^ and 10^6^ PFU of vGK5 intranasally 1 month earlier were challenged with 10^6^ PFU of VACV-WR by the i.n. route. Mice were monitored for weight loss for 5 days and organs were isolated to measure viral load in naïve and immunized mice. While naïve mice rapidly lost weight and appeared moribund on d5, mice immunized with both doses of vGK5 did not lose significant weight (Fig. 6A). Viral titers at day 5 post challenge were significantly reduced in the lungs (Fig. 6B) and ovaries (Fig. 6C) of mice immunized with 10^6^ and 10^4.5^ of vGK5 compared to unimmunized groups.

## Discussion

Using a murine model, we assessed the immunogenicity of a recombinant VACV that was engineered to lack a known virulence determinant of VACV, the N1L gene. Mice infected with sublethal doses of the attenuated vGK5 virus by the i.n., tail scarification, and i.p. routes had high frequencies of activated antigen-specific CD8 T cells in both mucosal and systemic sites. In addition, mice infected with vGK5 had levels of VACV-specific antibodies 3–5 months after immunization which were similar to levels elicited in mice immunized with VACV-WR. Our results indicate that the attenuated vGK5 virus, though more attenuated in vivo, is still immunogenic and able to elicit robust T as well as B cell responses.

The lung is the major portal of entry and transmission for variola virus, the etiologic agent of smallpox. Therefore it is critical to assess vaccination strategies that lead to robust cell mediated immunity in pulmonary as well as extrapulmonary tissues. VACV inoculation by the i.n. route has been used as a model that more closely approximates the route of infection with natural variola (smallpox) virus infections in humans; at high doses of VACV-WR i.n., mice develop fatal lung disease with high viral titers in lung tissue and the brain [6], [21]. Our data suggest that the N1L protein limits the strength and magnitude of the CD8 T cell immune response during acute intranasal VACV infections since deletion of the N1L gene allows mice infected with the attenuated virus to respond with a robust CD8 T cell response.

Recently two groups solved the structure of N1L which has striking homology to the Bcl-2 family of antiapoptotic genes [17], [18]. In vitro, the N1L protein inhibits NF-kB signaling after IL-1, TNF-α, LT-β, and TLR stimulation [15]. Under normal conditions, NF-kB is an antiapoptotic transcription factor and therefore inhibition of NF-kB signaling under these conditions could induce programmed cell death [25]. Replication of the N1L-deleted virus in cell culture has however been found to be indistinguishable from a wildtype as well as a revertant virus [13]. We hypothesize that the Bcl-2-like structure of N1L reconciles the observed lack of positive or negative effect on cell survival in vitro following N1L expression, with its otherwise fatal NF-kB inhibitory function.

Graham et al recently confirmed that transfected N1L DNA inhibited IL-1 and TRAF 6 signaling to NF-kB [26]. Vaccinia virus proteins A52 and B14 share a Bcl-2-like fold but have evolved to inhibit NF-kappaB rather than apoptosis. N1L appears to inhibit NF-kB dependent inflammatory cytokine production in mice, based on the observation that N1L-deficient vaccinia virus permits greater expression of NF-kB driven genes during *in vivo* VACV infection [16]. Furthermore, N1L also suppresses signaling to IRF3, more robustly than it does NF-κB. IRF3 signaling was not investigated by Cooray et al, although Bcl-2 family members also influence IRF3 signaling. Programmed cell death, IRF3 signaling and NF-kB signaling are three key pathways in the innate immune response, and Bcl-2 proteins, like N1L, are capable of inhibiting all three innate immune response pathways [27]–[32]. Since signaling via the innate immune system is thought to be involved in the adaptive immune response [33], [34], the N1L protein may contribute to impaired adaptive immune responses by inhibiting any combination of these innate signaling pathways.

In vivo, VACV-WR and vGK5 had different replication kinetics. In our studies, when equal doses of virus were administered after i.n. infection (10^3.5^ PFU), VACV-WR replicated to a 3–4 log higher titer compared to the vGK5 virus. Interestingly, when equal doses were administered by the i.p. route (10^6^ PFU), viral titers were not significantly different. Since the N1L protein was hypothesized to have an antiapoptotic function, increased survival of cells infected with VACV-WR which express the N1L protein versus cells infected with vGK5 virus could contribute to increased viral titers in the lungs after i.n. infection.

Intranasal infections with respiratory viruses result in the recruitment of virus-specific CD8+ T cell effectors in the lung during acute infection and persistence of these virus-specific T cells in the respiratory tract months after the infection has resolved [35]–[37]. Frequencies of antigen-specific T cells that are maintained in memory following virus infections are likely influenced by several factors including the amount of initial antigen available for T cell priming, viral replication in target tissues, the route of inoculation and the cytokine milieu. Virus titers in the lungs of mice infected with the attenuated N1L deleted virus by the i.n. route were several logs higher compared to lungs of mice infected with by the i.p. route. While frequencies of antigen-specific cells in the lungs during acute responses were not significantly impacted by these differences in viral loads, whether frequencies of B8R_20–27_ specific T cells are differentially maintained in memory is still unknown. Memory responses of these and other VACV-specific T cells therefore need to be further evaluated in mice infected with attenuated N1L deficient viruses.

Several factors including the initial antigen dose, the kinetics of virus replication in mucosal and systemic sites, the innate immune response, T cells as well as antibodies are likely to contribute to protection. Our data show that mice immunized with attenuated vGK5 virus by the intranasal route induced robust immunity and subsequently was able to protect mice from a lethal challenge with VACV-WR. The vGK5 virus is not currently a strain with satisfactory attenuation or safety profile and further clinical development would likely involve testing the effect of N1L inactivation in an established vaccine strain. We propose that the attenuated vaccinia virus lacking a major virulence gene N1L is an alternative that balances immunogenicity and safety. Our data have implications for the rational design of recombinant live vaccines against foreign antigens.

## Materials and Methods

### Cells

RMA murine lymphoma line (H-2^b^) was provided by Dr. Raymond M. Welsh, at the University of Massachusetts Medical School, Worcester, MA.

### Peptides

Peptides of VACV were based on published reports [20]. Peptides were synthesized at AnaSpec Inc. (San Jose, CA) and the Protein Chemistry Core Facility at the University of Massachusetts Medical School using an automated Rainin Symphony peptide synthesizer.

### Immunization of mice and preparation of splenocytes and lung lymphocytes

Female C57BL/6 (4–8 weeks old) mice were purchased from the Jackson Laboratories (Bar Harbor, ME). Mice were infected with 1×10^6^ PFU VACV-WR or vGK5 by the i.p. and tail scarification routes. For dermal scarification, 1×10^6^ PFU VACV-WR or vGK5 in 50 µl PBS was placed at the base of the tail and 10–20 scratches were made in a crosshatch pattern using a 21 gauge needle. For the i.n. infections, mice were anesthetized with isoflurane and 50 µl PBS containing the indicated dose of virus was instilled into the nares. This volume allows predominant but not exclusive installation intranasally into infected mice. Splenocytes and lung lymphocytes were collected at the indicated time points post-immunization. To isolate lymphocytes from the lungs of infected mice, lungs were minced and treated with 0.14 U/ml Blendzyme (Roche Diagnostics) and DNase I (Sigma Biochemicals) for 45 mins at 37 C. Cells were passed through a 40 micron cell strainer, lysed with RBC lysis buffer (Sigma) and resuspended in RPMI 1640 medium with 10% heat-inactivated fetal bovine serum (FBS) and 5×10^−5^ M 2-mercaptoethanol (2-ME). All mice were maintained in the Animal Facility at the University Of Massachusetts Medical School, which is regulated by AWA-1995, PHS-1986, MA140-1985, and following the AAALAC-1965 guidelines.

### Virus titration

Ovaries, lungs and spleens of infected mice were collected at the indicated time points and frozen at −80°C for virus titration. Briefly, organs were freeze- thawed 3 times in 0.5 ml MEM/2% FBS. 0.5 ml of a 0.25% trypsin solution was added to the tubes and they were incubated at 37°C for 30 mins. Organs were homogenized and dilutions of supernatants were added to confluent CV-1 cells in 6 well plates. After 48 hours media was removed and crystal violet added to the plates and plaques enumerated. Data shown indicate the number of plaque forming units (PFU) of vaccinia virus/gram of tissue in the lungs and spleen and PFU/ovary.

### ^51^Cr release assay

RMA cells were infected with VACV-WR or vGK5 (MOI = 5) for 18–20 hrs. Uninfected or virus-infected RMA target cells were then labeled with 0.25 mCi of ^51^Cr for 60 min at 37°C. Following labeling, the cells were washed three times and then resuspended in RPMI 1640 containing 10% FBS. Effector cells (ex vivo splenocytes or lung lymphocytes) were then added to virus-infected RMA cells in 96-well round-bottom plates at various effector∶target cell (E∶T) ratios. Plates were incubated for 4 hr at 37°C, supernatants were harvested (Skatron Instruments, Sterling, VA), and specific lysis was calculated as [(experimental release-spontaneous release)/(maximum release-spontaneous release)]×100. All assays were performed in triplicate. All experiments were performed at least twice. Spontaneous lysis was less than 15% in all assays.

### Tetramer and cell surface staining

The tetramer containing the immunodominant epitope of VACV-B8R_20–27_ was synthesized in the NIH Tetramer Facility. Splenocytes, lung cells and whole blood from infected and uninfected mice were initially stained with the viability marker LiveDead Aqua (Molecular Probes) for 30 mins. at 4°C. Cell suspensions were then washed in FACS buffer, blocked with CD16/CD32 mAb (Fc block 24G.2) (BD Biosciences, San Diego CA.) for 15 minutes at 4°C, stained with the PE conjugated B8R_20–27_ tetramer at room temperature for 30 mins. mAb directed at surface phenotypic markers CD3 (clone 145-2C11), CD8 (clone 53-6.7), CD11a (clone 2D7) and CD44 (clone 1M7) were added for 30 minutes at 4°C. Cells were washed and all cell preparations were fixed with Cytofix (BD Biosciences). Samples were analyzed on a FACSARIA flowcytometer. FlowJo (TreeStar Inc. Ashland, OR.) version 7.1 was used to analyze all the data.

### Enzyme-linked immunospot (ELISPOT) assay for single-cell IFN-γ secretion

ELISPOT assays were performed according to the manufacturer's protocol (Mabtech AB, Sweden) and as previously described [38]. Briefly, 96 well Multiscreen-IP plates (Millipore, Bedford, MA) were coated with 15 µg/ml of rat anti-mouse IFN-γ monoclonal antibody (AN-18) over night at 4°C. Then, freshly isolated splenocytes (2.5×10^5^/well) were incubated with the indicated peptides (1 µg/ml), or concanavalin A (ConA) (5 µg/ml) at 37°C for 18–20 hr in RPMI 1640 containing 10% FBS. Biotinylated rat anti-mouse IFN-γ monoclonal antibody (R4-6A2) was added and incubated for 2 hr at room temperature, followed by addition of streptavidin-horseradish peroxidase for 1–2 hr at room temperature. Spots were stained with Vector NovaREDTM Substrate kit for peroxidase (Vector Laboratories, Burlingame, CA). The precursor frequency was calculated as [(number of spots in experimental well - number of spots in medium control well)/total number of cells per well] ×10^6^. Experiments were performed in triplicate wells.

### Plaque Reduction Neutralization titers

Serial two fold dilutions of heat inactivated sera from infected mice were incubated with VACV-WR for 60 minutes at 37c. Following the incubation, 0.25 mls of the virus/antibody mixture, virus alone or media was added to appropriate wells of confluent BSC-40 cells cultured in MEM. After 48 hours media was removed and crystal violet added to the plates and plaques enumerated. Titers were defined as the reciprocal serum dilution that caused a 50% reduction in viral plaques (PRNT50).

### Statistical analysis

Statistical significance of the data was determined by using Student's *t* test.
